# POT1 inhibits the efficiency but promotes the fidelity of nonhomologous end joining at non-telomeric DNA regions

**DOI:** 10.18632/aging.101339

**Published:** 2017-12-08

**Authors:** Yang Yu, Rong Tan, Qian Ren, Boya Gao, Zhejin Sheng, Juanlian Zhang, Xiaoqing Zheng, Ying Jiang, Li Lan, Zhiyong Mao

**Affiliations:** ^1^ Clinical and Translational Research Center of Shanghai First Maternity & Infant Hospital, Shanghai Key Laboratory of Signaling and Disease Research, School of Life Science and Technology, Tongji University, Shanghai 200092, China; ^2^ State Key Laboratory of Natural Medicines, China Pharmaceutical University, Nanjing, China; ^3^ University of Pittsburgh Cancer Institute, University of Pittsburgh School of Medicine, Pittsburgh, PA 15213, USA; ^4^ Center for Molecular Medicine, Xiangya Hospital, Central South University, Changsha, Hunan 410008, China

**Keywords:** POT1, DNA double strand break repair, NHEJ fidelity, NHEJ efficiency, Artemis, DNA Lig3

## Abstract

Robust DNA double strand break (DSB) repair and stabilized telomeres help maintain genome integrity, preventing the onset of aging or tumorigenesis. POT1 is one of the six factors in the shelterin complex, which protects telomeres from being recognized as DNA damages. TRF1 and TRF2, two other shelterin proteins, have been shown to participate in DNA DSB repair at non-telomeric regions, but whether POT1, which binds to single strand telomeric DNA at chromosomal ends, is involved in DNA DSB repair has not been assessed. Here we found that POT1 arrives at DNA damage sites upon the occurrence of DNA DSBs. It suppresses the efficiency of nonhomologous end joining (NHEJ), the major pathway for fixing DNA DSBs in mammals, but surprisingly promotes NHEJ fidelity. Mechanistic studies indicate that POT1 facilitates the recruitment of Artemis, which is a nuclease and promotes fidelity of NHEJ, to DNA damage sites. In addition, we found that overexpression of POT1 inhibits the protein stability of Lig3, which is the major regulator of alternative NHEJ (alt-NHEJ), therefore suppressing the efficiency of alt-NHEJ. Taken together we propose that POT1 is a key factor regulating the balance between the efficiency and fidelity of NHEJ at non-telomeric DNA regions.

## INTRODUCTION

Genomic stability is constantly threatened by DNA damages arising from a variety of endogenous and exogenous sources. Among all types of DNA damages, DNA double strand breaks (DSBs) are the most harmful as unrepaired or inappropriately repaired DSBs may introduce deletions, insertions or chromosomal rearrangements to genomes, leading to severe consequences including the onset of aging and tumorigenesis. Therefore, precisely repairing DNA DSBs is critical to stabilizing genomes. Two repair pathways, non-homologous end joing (NHEJ) and homologous recombination (HR), are responsible for mending the broken ends [1-3]. NHEJ can be further categorized into two sub-pathways, conventional NHEJ (c-NHEJ) and alternative NHEJ (alt-NHEJ) [2, 4]. The factors participating in c-NHEJ process includes Ku70-Ku80 heterodimer, DNA-PKcs, Artemis, and XLF-XRCC4-Lig4 complex. The molecular mechanisms of alt-NHEJ pathway have not been well studied, but several factors including PARP1 and DNA Lig3 have been documented to be involved in the repair process [5]. Although HR directed repair is an error-free process if strictly regulated as it copies the missing information directly from the identical sister chromatids, it occurs only in S/G2 phase and accounts for merely one quarter of successfully repaired events in mammalian cells [6-8]. On the contrary, the inaccurate NHEJ is the major pathway for repairing DNA DSBs, fixing the remaining 75% of DNA DSBs in rapidly dividing cells [6, 7]. Much knowledge has been learned on the regulatory mechanisms of DNA DSB repair by NHEJ [9-12], but most of the studies have been on how the efficiency of NHEJ is determined by various factors. Surprisingly and intriguingly, the regulation of NHEJ fidelity has only received little attention.

POT1 (Protection of Telomere 1), a well-characterized factor bound to telomeric single-stranded DNA [13], caps the chromosomal ends by tethering to TRF1 and TRF2 to form the T-loop structure [14], therefore preventing DNA ends from being recognized as DSBs. Loss of POT1 destabilizes genomes by causing massive end-to-end fusions [15]. Mutations in POT1 are often associated with a variety of types of cancers [16]. Mechanistic studies indicate that at telomeric regions POT1 suppresses the activation of ATR (Ataxia telangiectasia and Rad3 related) and the alternative NHEJ pathway [17, 18]. Intriguingly, several previous reports have indicated that two essential shelterin proteins TRF1 and TRF2 are recruited to DNA DSB sites to promote DNA DSB repair by homologous recombination at non-telomeric regions [19-22]. In addition, a recent report indicates that biochemically POT1 not only binds to telomeric TTAGGG repeats but also non-telomeric DNA [23], strongly suggesting that POT1 may also participate in DNA DSB repair at non-telomeric regions.

In this study we studied whether and how POT1 participates in DNA DSB repair. We found that POT1 may rapidly arrive at DNA damages. At DNA damage sites, it promotes the fidelity of NHEJ while inhibits the NHEJ efficiency. Mechanistic studies indicate that POT1 facilitates the recruitment of Artemis to promote NHEJ fidelity while POT1 overexpression inhibits the protein stability of Lig3 to suppress NHEJ efficiency.

## RESULTS

### POT1 is rapidly recruited to nontelomeric DNA damage sites

**Figure 1:**
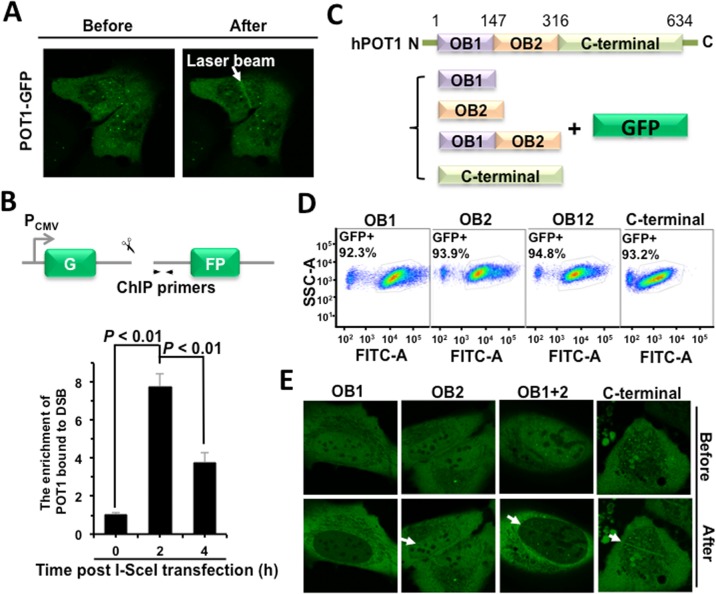
POT1 is rapidly recruited to nontelomeric DNA damage sites (**A**) POT1 is recruited to DNA damage sites within 1-second post microirradiation. U2OS cells were microirradiated to generate DSBs in a line pattern using a 405 nm diode laser. (**B**) Diagram of the site for which ChIP primers were designed (arrows). At different time points post I-SceI transfection, NHEJ-I9a cells were harvested and lysed for ChIP assay with an antibody against POT1, followed by quantitative PCR analysis. The procedure for ChIP is as previously described [12]. (**C**) Schematic depiction of different domains of POT1 tagged with GFP. (**D**) Comparable expression of GFP-tagged different domains of POT1. The U2OS cells were transfected with different amounts of vectors encoding OB1 (0.67 μg), OB2 (0.5 μg), OB12 (1 μg), C-terminal (3 μg). At 48 h post transfection, cells were harvested for FACS analysis. **(E)** Analysis of recruitment of different POT1 domains.

To test the hypothesis that POT1 participates in DNA DSB repair, we first examined if POT1 is recruited to DNA DSB sites using microirradiation. We found that GFP-tagged POT1 is rapidly recruited to DNA damage sites in a human bone osteosarcoma epithelia cell line U2OS (Figure 1A), while GFP itself is not recruited to DNA lesions (Figure S1). To confirm POT1 arrives at DNA DSB sites, we performed ChIP assay using our well-established NHEJ-I9a cell line with one copy of NHEJ reporter integrated into genomes [6]. We found that POT1 was recruited to a defined DNA DSB site at 2 h post I-SceI transfection (Figure 1B), when DNA DSBs were induced within 30 minutes [6, 12]. Taken together, we demonstrated that POT1 is rapidly recruited to DNA DSB sites.

The full-length POT1 comprises three domains: two OB-fold (oligonucleotide/oligosaccharide-binding fold) domains and a C-terminal domain [13]. Both OB domains bind to the 3′ single strand overhang, but OB1 has higher affinity to single strand telomeric DNA than OB2 does. The C-terminal domain contains a NLS (nuclear localization sequence) and is also critical for the interaction with TPP1, another shetlerin factor. To determine which domain is required for the recruitment of POT1 to DNA damage sites, we created vectors encoding GFP tagged different domains of POT1 (Figure 1C). We first optimized plasmids amount for transfection to equalize the expression (Figure 1D). Using the same assay, we found that both OB2 and C-terminal rather than OB1 domain may successfully come to damage sites (Figure 1E).

### POT1 promotes NHEJ fidelity but inhibits NHEJ efficiency

**Figure 2:**
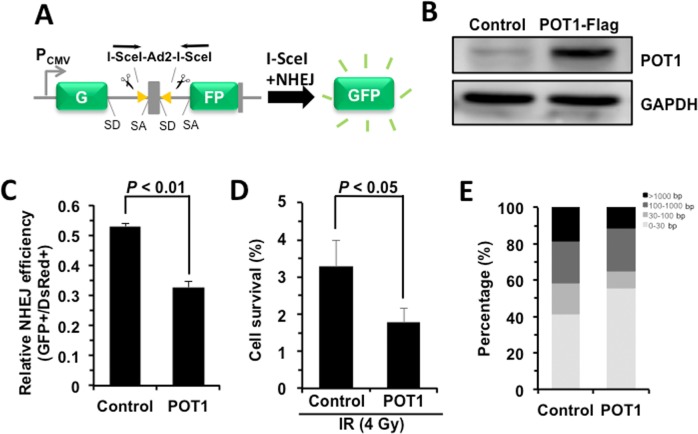
POT1 promotes NHEJ fidelity but inhibits NHEJ efficiency (**A**) Schematic picture of NHEJ reporter cassette. The reporter and the cell line harboring it are as previously described [7, 24]. (**B**) Expression of FLAG-tagged POT1. (**C**) The effect of POT1 overexpression on NHEJ efficiency. The NHEJ-I9a was transfected with POT1 vector, I-SceI vector and DsRed for normalizing transfection efficiency using Lonza 4D machine. On day 3 post transfection, cells were harvested for FACS analysis. (**D**) Overexpressing POT1 sensitizes HCA2-hTERT cells to X-Ray. POT1 overexpressing cells were treated with X-Ray at 4 Gy, and then harvested, reseeded to plates at different numbers. On day 14 post IR, cells were stained with Commassie solution and colonies with at least 50 cells were counted. Cell survival was calculated as the ratio of the relative plating efficiencies of X-Ray treated versus control cells. (**E**) Analysis of NHEJ fidelity. The method is as previously reported [25]. At least forty clones were used for junction sequencing. bp: base pairs.

To understand the role of POT1 in non-telomeric DNA DSB repair, we tested how POT1 affects NHEJ and HR efficiency using our GFP-reporter cell lines [7, 24] (Figure 2A). We found that, similar to TRF2 [22], overexpressing POT1 significantly suppresses NHEJ by 40% but has very mild effect on HR (Figure 2, B and C, Figure S2). To rule out the possibility that POT1 inhibits NHEJ efficiency through altering cell cycle distribution, we analyzed cell cycle distribution in cells with POT1 overexpressed. We found that POT1 overexpression has no significant effect on cell cycle (Figure S3). We also examined the functional consequences of POT1 overexpression. We found that as a consequence of reduced NHEJ efficiency with POT1 overexpressed in cells, overexpression of POT1 suppresses the clearance of γH2AX, a marker for DNA double strand breaks, in response to IR (Figure S4, A and B). Moreover, in consistence with its inhibitory effect on NHEJ efficiency, we found that overexpressing POT1 sensitizes cells to ionizing radiation at a dosage of 4 Gy (Figure 2D). However, we did not observe significant change of apoptotic rates of cells in the presence of POT1 overexpression (Figure S4C), suggesting that cells with unrepaired DNA DSBs probably entered senescence rather than apoptosis.

We then assessed the NHEJ fidelity in the presence of POT1 overexpression using our well-established assay for analyzing the fidelity [25]. We created DNA DSBs on our NHEJ reporters by *in vitro* I-SceI digestion. The linearized NHEJ reporters were introduced to HCA2-hTERT cells together with POT1 or a control vector. Then repaired NHEJ constructs were rescued by DNA extraction and *E.Coli* transformation. The junctions of the rescued plasmids were sequenced and the NHEJ fidelity was then determined by comparing the deletion size at repaired sites [25]. Surprisingly we found that the average deletion size is dramatically reduced by ~46% with POT1 overexpressed (245 bp vs 450 bp), strongly indicating that POT1 is a critical regulator of the balance between the efficiency and fidelity. To precisely compare the fidelity between control and POT1 overexpression groups, we grouped rescued clones into four different classes. We found the percentage of clones with deletions larger than 1000 bp is reduced from 18.3% to 11.1% in POT1 over-expressing cells, while the percentage of clones with deletions less than 30 bp is increased from 41.7% to 55.5% in the presence of POT1 overexpression (Figure 2E).

### POT1 promotes NHEJ fidelity by facilitating the recruitment of Artemis to DNA damage sites

**Figure 3:**
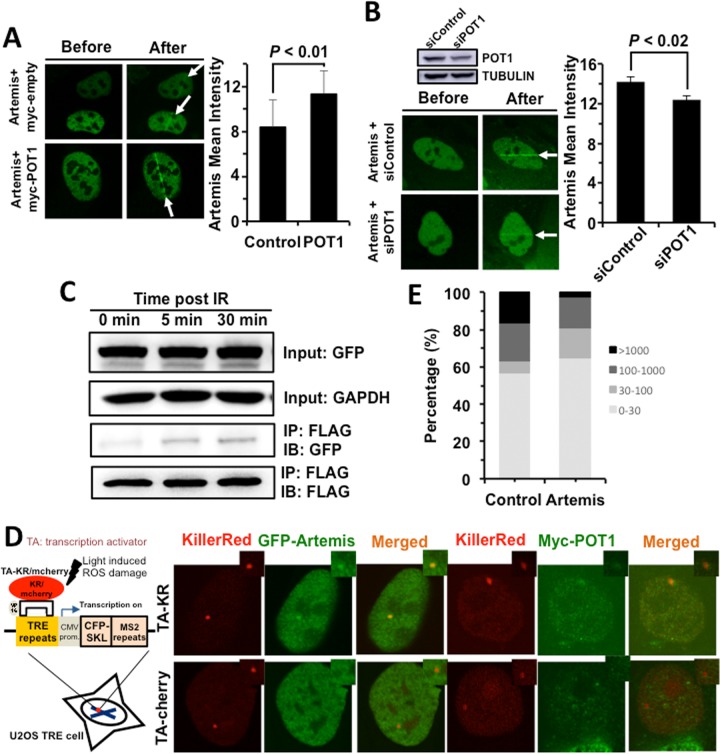
POT1 stimulates the recruitment of Artemis to DNA damages sites (**A**) POT1 overexpression stimulates the recruitment of Artemis to laser induced DNA damage sites. The recruitment of Artemis is quantified using the software of Leica LAS AF Lite. (**B**) Mildly knocking down POT1 significantly suppresses the recruitment of Artemis to DNA lesions induced by lasers. The recruitment of Artemis is quantified using the software of Leica LAS AF Lite. (**C**) POT1 interacts with Artemis upon DNA damages. 293FT cells were co-transfected with a plasmid encoding POT1-FLAG and a vector encoding Artemis-GFP. On day 1 post transfection, cells were irradiated with X-Ray at 6 Gy. At different time points, cells were harvested for immunoprecipitation with an antibody against FLAG, followed by Western blot analysis. (**D**) Both POT1 and Artemis are recruited to a given DNA damage site. The KillerRed (KR) system is as previously described (25). In brief, it is a fluorescent protein derived from hydrozoan. Long exposure of cells expressing the KR protein generates ROS-induced DNA DSBs. The U2OS reporter cell line harboring ~ 200 copies of TRE elements was co-transfected with myc-POT1 or GFP-Artemis and TA (transcription activator) –KR or TA-cherry. Both TA-KR and TA-cherry proteins may recognize the TRE elements. TA-KR causes DNA damages at the given site while TA-cherry is utilized as a negative control. The transfected cells were then exposed to light, followed by fixation and immunostaining for further analysis. (**E**) The effect of Artemis overexpression on NHEJ fidelity. The analysis of NHEJ fidelity is as described in Figure 2E.

We then set out to understand the regulatory mechanisms of NHEJ by POT1. POT1 arrives at DNA damage sites within 1 second, indicating that it affects NHEJ fidelity at early stage. For conventional NHEJ (c-NHEJ), the early step affecting NHEJ fidelity might be the end processing regulated by nucleases including Artemis, MRE11 and CtIP. We therefore examined how POT1 affects the recruitment of Artemis, MRE11 and CtIP, and found that POT1 overexpression significantly stimulates the recruitment of Artemis, but neither MRE11 nor CtIP (Figure 3A and Figure S5). In addition, we examined the recruitment of Artemis to DNA damage sites in U2OS cells with POT1 mildly knocked down. We found that partially knocking down POT1 significantly suppresses the recruitment of Artemis to DNA damage sites (Figure 3B).

To further confirm whether POT1 is associated with Artemis, we performed co-IP experiments. We found POT1 interacts with Artemis but not other NHEJ factors and the interaction is enhanced upon IR (Figure 3C, Figure S6). We also confirmed that both Artemis and POT1 are recruited to a given DNA double strand break site using the KillerRed (KR) reporter system (Figure 3D) [26], indicating the two factors interact with each other at DNA DSB sites.

However, surprisingly, whether Artemis improves NHEJ fidelity has not been characterized. We then tested its effect on NHEJ fidelity. We found over-expressing Artemis greatly improves NHEJ fidelity. The average deletion size of rescued plasmids is ~ 3-fold lower in Artemis overexpressing cells than that in control cells (124 bp vs 339 bp). The percentage of clones with deletion size larger than 1000 bp is reduced from 16.7% to 2.7% in Artemis overexpressing cells while the percentage of clones with deletion size smaller than 30 bp is increased from 56.7% to 64.9% (Figure 3E). Moreover, we analyzed how Artemis affects the change of NHEJ fidelity in HCA2-hTERT cells with POT1 mildly depleted (Figure S7A). We found that partially knocking down POT1 compromised the stimulatory effect of Artemis on NHEJ fidelity. In cell with POT1 mildly depleted, the average deletion size of rescued plasmids is ~ 1.88 fold lower in Artemis overexpressing cells than that in control cells (177 bp vs 332 bp). The percentage of clones with deletion size larger than 1000 bp is only reduced from 15.8% to 8.6% (Figure S7B).

Taken these results together, we conclude that POT1 facilitates the recruitment of Artemis to DNA damage sites to improve NHEJ fidelity.

### POT1 inhibits the expression of Lig3

**Figure 4:**
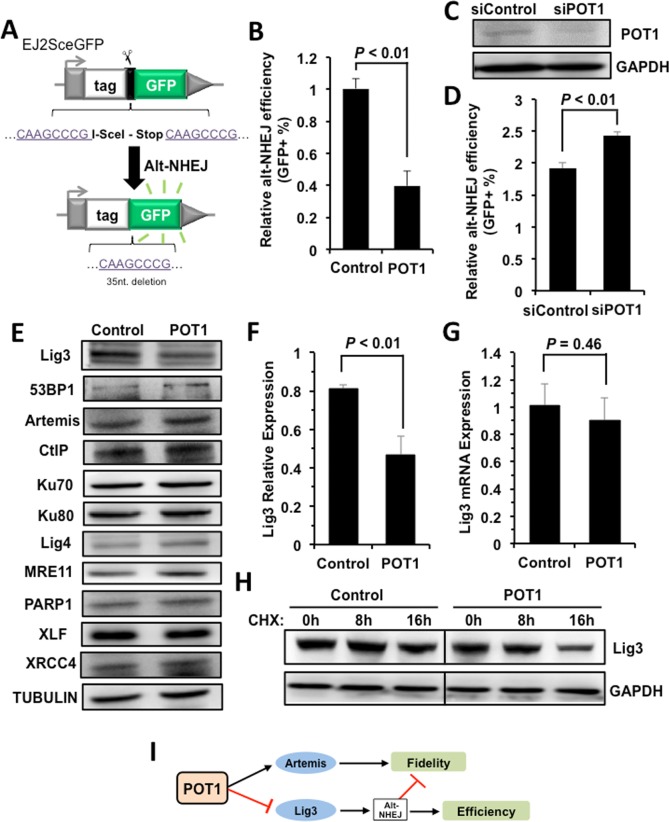
POT1 inhibits alt-NHEJ efficiency and promotes the degradation of Lig3 (**A**) Schematic diagram of EJ2-GFP for analyzing the alt-NHEJ efficiency. The mechanism of the reporter cassette is as previously described [6]. (**B**) Overexpression of POT1 inhibits alt-NHEJ efficiency. The reporter construct was digested with I-SceI restriction enzyme *in vitro*, followed by being transfected to HCA2-hTERT cells together with a control vector or a plasmid encoding POT1. On day 3 post transfection, cells were harvested for FACS analysis. (**C**) and (**D**) Mildly knocking down POT1 in HeLa cells significantly stimulates the alt-NHEJ efficiency. HeLa cells were transfected with siRNA against POT1 twice with two days interval, followed by a transfection of I-SceI linearized EJ2-GFP reporter. On day 3 post transfection, cells were harvested for FACS analysis. (**E**) Expression of important NHEJ factors in the absence or presence of POT1 overexpression. (**F**) Quantification of Lig3 expression using ImageJ software. The relative expression of Lig3 is calculated as the ratio of Lig3 expression versus TUBULIN. (**G**) Lig3 expression was not affected at transcriptional level in POT1 overexpressing cells. At 24 h post POT1 transfection, cells were harvested for mRNA extraction. Then Quantitative PCR analysis was performed with primers indicated. The primers used for q-PCR of Lig3 are as follows: Forward: 5′- TATGGCACGGGACCTAG -3′, Reverse: 5′- CTGTTGCTGCTCATCCTC -3′. The primers used for q-PCR of GAPDH are as follows: Forward: 5′ATGACATCAAGAAGGTGGTG3′, Reverse: 5′CATACCAGGAAATGAGCTTG3′. The transcript level of Lig3 was determined using delta CT method [38]. (**H**) POT1 overexpression promotes Lig3 degradation. 293FT cells with a control vector or a vector encoding POT1 transfected were treated with cycloheximide (CHX) at 50 μg/ml. At different time points post the treatment, cells were harvested for Western blot analysis. (**I**) The model of POT1 regulating DNA DSB repair at non-telomeric regions.

However, the stimulated recruitment of Artemis by POT1 cannot explain why POT1 represses NHEJ efficiency. We hypothesize that POT1 affects another mechanistically distinct and exclusively mutagenic NHEJ pathway – alt-NHEJ [17]. We employed a GFP gene based reporter cassette (Figure 4A), which measures the efficiency of alt-NHEJ [27], to test if POT1 inhibits alt-NHEJ. We found that overexpressing POT1 suppresses alt-NHEJ efficiency (Figure 4B), while mildly knocking down endogenous POT1 significantly improves alt-NHEJ (Figure 4, C and D). These results strongly suggest that POT1 inhibits NHEJ efficiency by suppressing alternative NHEJ pathway. In addition, suppressing more error-prone alt-NHEJ by POT1 also contributes to promoting the fidelity of NHEJ.

To understand the regulatory mechanisms, we examined the expression change of major NHEJ factors in cells with POT1 overexpressed, and found that the expression of Lig3, which is a major alt-NHEJ factor, is suppressed in POT1 overexpressing cells (Figure 4, E and F). Interestingly we did not observe the decline of Lig3 mRNA level in cells overexpressing POT1, indicating that the impaired expression of Lig3 is at post-transcriptional level (Figure 4G). Indeed, in POT1 overexpressing cells treated with CHX, a drug blocking protein synthesis, the protein level of Lig3 is dramatically reduced in comparison to that in control group cells (Figure 4H).

Collectively our mechanistic studies indicate that POT1 promotes fidelity but inhibits efficiency of NHEJ through stimulating the recruitment of Artemis to damage sites, and negatively regulating Lig3 expression at the protein level (Figure 4I).

## DISCUSSION

Impaired DNA repair capacity and destabilized telomeres contribute to the rise of genomic instability [28, 29]. Both of the factors are major hallmarks of aging and the onset of tumorigenesis in mammals [30-32]. Intriguingly, the crosstalk between DNA double strand break repair machinery and the shelterin complex for stabilizing genomes has long been an interesting topic in the field of genomic stability. On one hand, various DNA DSB repair factors such as DNA-PKcs, Ku70/80 heterodimer, MRE11, Rad50, Rad51D, BRCA1, PARP1 are present at telomeres. Unlike their functions of promoting DNA repair at non-telomeric regions, these factors coordinate with shelterin proteins to stabilize the T-loop structure and suppress DNA repair at telomeric DNA regions [33, 34], therefore protecting chromosomal ends from aberrant end-to-end fusions. However, on the other hand, although several reports suggest that shelterin proteins including TRF1 and TRF2 are recruited to broken ends and participate in DNA DSB repair at non-telomeric DNA regions [19-22], their roles in non-telomeric DNA lesions are still under debate, largely due to unknown functions of the two telomere binding factors in non-telomeric DNA DSB repair. Our report of POT1 participating in the regulation of NHEJ fidelity unravel the function of a telomere binding protein in DNA DSB repair, further confirming that similar to the protective function of DNA repair protein at chromosomal ends the shelterin proteins also play positive roles in maintaining genome integrity by promoting DNA repair at non-telomeric DNA regions. Nevertheless, whether POT1 regulates NHEJ fidelity by cooperating with the other two telomeric binding factors TRF1 and TRF2 needs to be further investigated.

Another potential obstacle that needs to be overcome during the investigation of function of telomere binding proteins in DNA repair at non-telomeric DNA regions is that simply depleting these shelterin factors may cause cell cycle arrest by triggering DNA damage response due to the loss of T-loop structure at chromosomal ends [35, 36]. In this study we mainly performed over-expression experiments to avoid any potentially confusing results. However, to ultimately elucidate the regulatory mechanisms of POT1 on NHEJ fidelity, creating a POT1 mutant, which abolishes its DNA repair capability but retains its normal function of stabilizing telomeres by forming the T-loop structure together with other five member of shelterin proteins, would help advance our understanding of the function of POT1 as a DNA repair factor.

Repairing DNA DSBs by NHEJ has been studied by numerous groups from different perspectives in various contexts, but most of the research has been focused on the regulation of NHEJ efficiency. Not much attention has been paid to the regulatory mechanisms of NHEJ fidelity. Using our well-established assay for analyzing the NHEJ fidelity, we demonstrated that surprisingly POT1 is a critical regulator of this process. We speculate that on one hand the rapidly recruited POT1 at DNA damage sites might help prevent the recruitment of alt-NHEJ factors such as PARP1 to broken ends. On the other hand, it is possible that POT1, which binds to 3′ single strand overhang on chromosomal ends [13], also has high affinity to non-telomeric single strand DNA overhang, therefore facilitating the recruitment of Artemis to DNA lesions to promote the fidelity. Moreover, POT1 may participate in the process of destabilizing Lig3 to inhibit the highly mutagenic alt-NHEJ, which on one hand leads to a reduction of NHEJ efficiency, on the other hand, forces cells to choose less error-prone c-NHEJ, therefore promoting the repair fidelity. Nevertheless, how POT1 promotes the degradation of Lig3 protein needs to be further investigated. Since we observed the reduced protein level of Lig3 with POT1 overexpressed in the absence of DNA damages, we may rule out the possibility that POT1 promotes its degradation at damage sites. We hypothesize that POT1 may promote the nuclear export and proteasomal degradation of Lig3 by activating kinases involved in DNA damage responses such as ATM-CHK2 or ATR-CHK1. As a result of reducing Lig3 expression, POT1 prevents the aberrant DNA fusion at chromosomal ends and inhibits the alternative NHEJ efficiency at non-telomeric DNA DSBs.

In summary, our study for the first time demonstrates that POT1 as a well-known telomere binding protein is also a critical factor participating in DNA DSB repair at non-telomeric DNA regions. We propose that POT1 is an essential factor regulating the balance of NHEJ efficiency and fidelity.

## METHODS

### Cell culture

The immortalized neonatal foreskin fibroblast HCA2-hTERT and its derived cell lines including NHEJ-I9a and HR-H15c were cultured in MEM (Mediatech, Cat. # 10-010-CVR) containing 10% FBS (ScienCell, Cat. # 0510) supplemented with 1% penicillin/streptomycin (Gibco, Cat. # 15140-122) and 1% nonessential amino acids (Gibco, Cat. # 11140-050). U2OS cell lines were cultured in DMEM media (Mediatech, Cat. # 10-013-CVR) containing 10% FBS supplemented with 1% penicillin/streptomycin. All cultured cells were maintained in incubators with 5% CO_2_ and 3% O_2_ at 37°C and counted by a Countstar machine (Model: IC1000, China).

### Laser microirradiation

For laser irradiation, the Olympus FV1000 confocal microscopy system (Cat. # F10PRDMYR-1, Olympus) with FV1000 SIM Scanner and 405 nm laser diode (Cat. # F10OSIM405, Olympus) was employed. Cells were incubated at 37°C on a thermo-plate (MATS-U52RA26 for IX81/71/51/70/50; metal insert, HQ control, Cat. # OTH-I0126) in Opti-MEM during observation to avoid pH changes. Before irradiation, cells were treated with 100 μM 8-MOP, a photosensitizer, for 5-10 min [37]. For inducing DNA damage, a 405 nm laser was used with the indicated power; the output power of the 405 laser passed through the lens is 5 mW/scan. Laser light was passed through a PLAPON 60x oil immersion objective lens (super chromatic abe. corr. obj W/1.4NA FV, Cat. # FM1-U2B990). At least ten cells were irradiated in every experiment and FV1000 software was used for acquisition of images.

### Immunofluorescence and microscopy

Cells were grown on the glass bottom dishes (MatTekCo.) and co-transfected with plasmids myc-POT1/GFP-artemis and TA-KR/TA-cherry. After 36-48 hours growth, cells were applied under light for 25 minutes and 30 minutes for recovery. Then, cells were washed three times with PBS and fixed with 4% paraformaldehyde for 15 minutes at room temperature. After 15 minutes, cells were washed with PBS for 5 minutes on the rotating bed and permeablilzed using 0.2% Triton X-100 for 10 minutes. Then by using the 5% BSA (Sigma), cells were blocked for 1 hour at the room temperature. Primary antibody was diluted (1:1000) and applied to the cells that transfected with myc-POT1 at 4°C overnight. Cells were washed for five minutes and three times repeats with PBST (0.05% Tween-20 in PBS) after the primary antibody incubation. An Alexa Fluor-488-conjugated secondary antibody (Thermo Fisher Scientific) was applied to the cells for 1 hour in the dark at room temperature. Cells were then washed for 5 minutes with PBST and stained with DAPI for 10 minutes, washed for 5 minutes again with PBST after the DAPI staining. Cells were imaged under the Olympus FV1000 confocal microscopy system (Cat. F10PRDMYR-1, Olympus).

### Transfections

NHEJ-I9a or HR-H15c cell lines with stably integrated NHEJ or HR reporter cassettes were seeded at a density of 1×10^5^ cells/well on a 6-well plate, and cultured for 48 hours before electroporation. Cells were transfected with 1.67 μg vectors encoding POT1 or a pcDNA3.1 control plasmid, 1.67 μg vectors encoding I-SceI and 0.02 μg DsRed-N1 as the internal control.

For NHEJ fidelity analysis, HCA2-hTERT cells were seeded at a density of 5×10^5^ cells on a 10 cm dish, cultured for 48 hours before electroporation. NHEJ reporter cassettes plasmids were linearized by I-SceI restriction enzymes (NEB, Cat. # R0694L) and purified by a DNA purification kit (QIAGEN, Cat. # 20051). Exponentially growing cells were transfected with 0.8 μg of the NHEJ reporter construct, together with 5 μg vectors encoding POT1 or pcDNA3.1 plasmids using a Lonza 4D machine with DT-130 program.

For U2OS cells, transfections were performed using FuGENE6 reagent (Promega, Cat. # E2691).

For Hela cells, siControl and siPOT1 RNA was transfected using Lonza 4D machine with CN-114 program twice with a 48 h interval. The targeting sequence against POT1 was: 5′- GTACTAGAAGCCT ATCTCA -3′.

### FACS analysis

On day 3 post transfection, cells were harvested for FACS analysis on FACS verse (BD Biosciences, USA). At least 20,000 cells were counted for calculating NHEJ or HR efficiency. All the data was further analyzed using the software of FlowJo.

### Plasmid rescue

At 48 h post transfection of 0.6 μg of linearized NHEJ construct into 10^6^ cells, cells were harvested for extracting all DNA including the repaired NHEJ substrate. Then 0.8 μg extracted DNA was transformed to 50 μl competent *E.Coli* cells (Takara, Cat # 9021). The rescued NHEJ plasmids were isolated and sequenced using two primers at the upstream and down-stream of two I-SceI recognition sites: 5′GACCACTGGATTCAGAAGCGATC3′, 5′ATGGTA TCATTTTCGGTAGC3′. The sequencing service was provided by Genewiz (Shanghai, China). At least forty plasmids were sequenced followed by analysis using a DNA Strider software.

### Co-immunoprecipitation

The irradiated 293FT cells were harvested and lysed in IP lysis buffer at different time points post IR treatment using an X-Ray machine (RX-2000pro 160kv, Radsource, US). The lysates were then sonicated at 10% power for 2 minutes twice, followed by centrifugation for 10 minutes at 13,000 rpm at 4°C. The supernatants were pre-cleared by incubating them with 20 μl protein A/G agarose (Santa Cruz, #sc-2003) and IgG (Abmart, #b30010M) for 1 hour at 4°C. The pre-cleared supernatants were then incubated with antibodies for overnight at 4°C, followed by incubation with protein A/G sepharose for 2 h. Then the mixtures were centrifuged for 5 minutes at 2,500 rpm at 4°C and washed 3 times with cold PBS. The precipitated proteins were released by boiling in 2 × sample buffer. Afterwards, Western blot was performed for further analysis.

### Western blot

After 48 hours post transfection, cells were harvested for protein extraction. Thirty μg protein extracts were loaded onto 10% SDS-PAGE gels followed by Western blot analysis. The information about antibodies is as follows: POT1 (Abcam, Cat. # ab21382), FLAG (Abclonal, Cat. # AE005), Artemis (Abclonal, Cat. # A5615), Lig3 (Abcam, Cat. # ab587), 53BP1 (CST, Cat. # 4937S), CtIP (Active motif, Cat. # 61141), Ku70 (Abclonal, Cat. # A0883), Ku80 (Abclonal, Cat. # A5862), Lig4 (Abclonal, Cat. # A1743), MRE11 (Abclonal, Cat. # A2559), PARP1 (Abclonal, Cat. # A3121), XLF (Abclonal, Cat. # A4985), XRCC4 (Abclonal, Cat. # A7539), β-TUBULIN (CMCTAG, Cat. # AT0050), ACTIN (Abclonal, Cat. # AC006), γH2AX (CST, Cat. # 9718).

## SUPPLEMENTARY MATERIALS FIGURES


