# Supplementary material for: POT1 inhibits the efficiency but promotes the fidelity of nonhomologous end joining at non-telomeric DNA regions
Source: Aging (Albany NY). 2017 Dec 8;9(12):2529–43. doi: 10.18632/aging.101339 (PMC5764391; doi:10.18632/aging.101339)
Supplement: Supplementary file 1 [file aging-09-2529-s001.pdf]

SUPPLEMENTARY MATERIAL

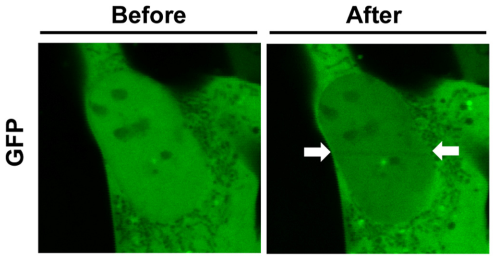

Supplementary Figure 1 . GFP is not recruited to DNA damage sites. U2OS cells transfected with GFP were microirradiated to generate DSBs in a line pattern using a 405 nm diode laser.

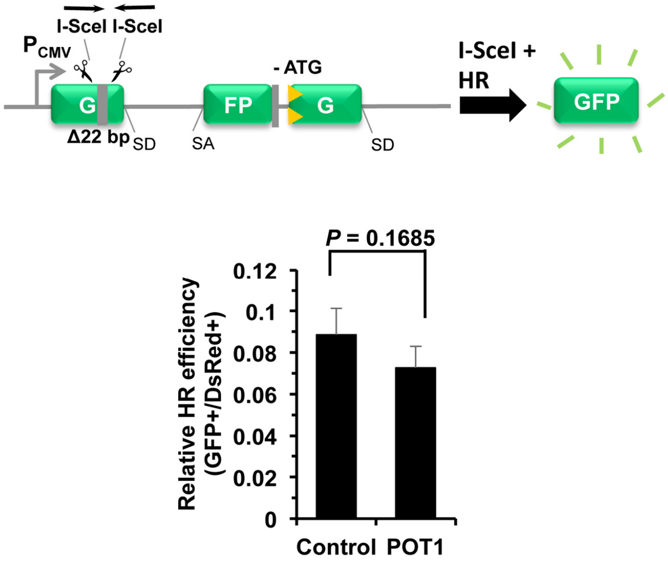

Supplementary Figure 2. POT1 overexpression has no significant effect on HR. The HR reporter was as previously reported (Mao et al. 2007). 1×105 rapidly growing reporter cell line HR-H15C was transfected with 1.67 μg I-SceI vector and 0.02 μg vector encoding DsRed for normalizing transfection efficiency together with a control vector or POT1 expression vector using Lonza 4D machine. On day 3 post transfection, cells were harvested for FACS analysis.

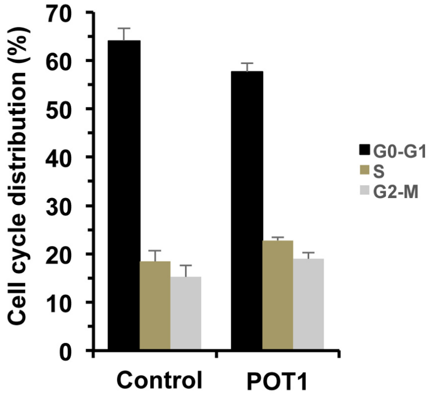

Supplementary Figure 3. POT1 overexpression has no significant effect on cell cycle distribution. HCA2-hTERT cells were transfected with a control vector or a POT1 expressing vector. At 24 h post trans-fection, cells were harvested for fixation with 70% ethanol stored at -20 °C. The fixed cells were incubated at 4 °C for at least 24 h before being proceeded for PI staining and FACS analysis on FACSverse. At least 10,000 events were collected in each analysis. Data was further analyzed by FlowJo software.

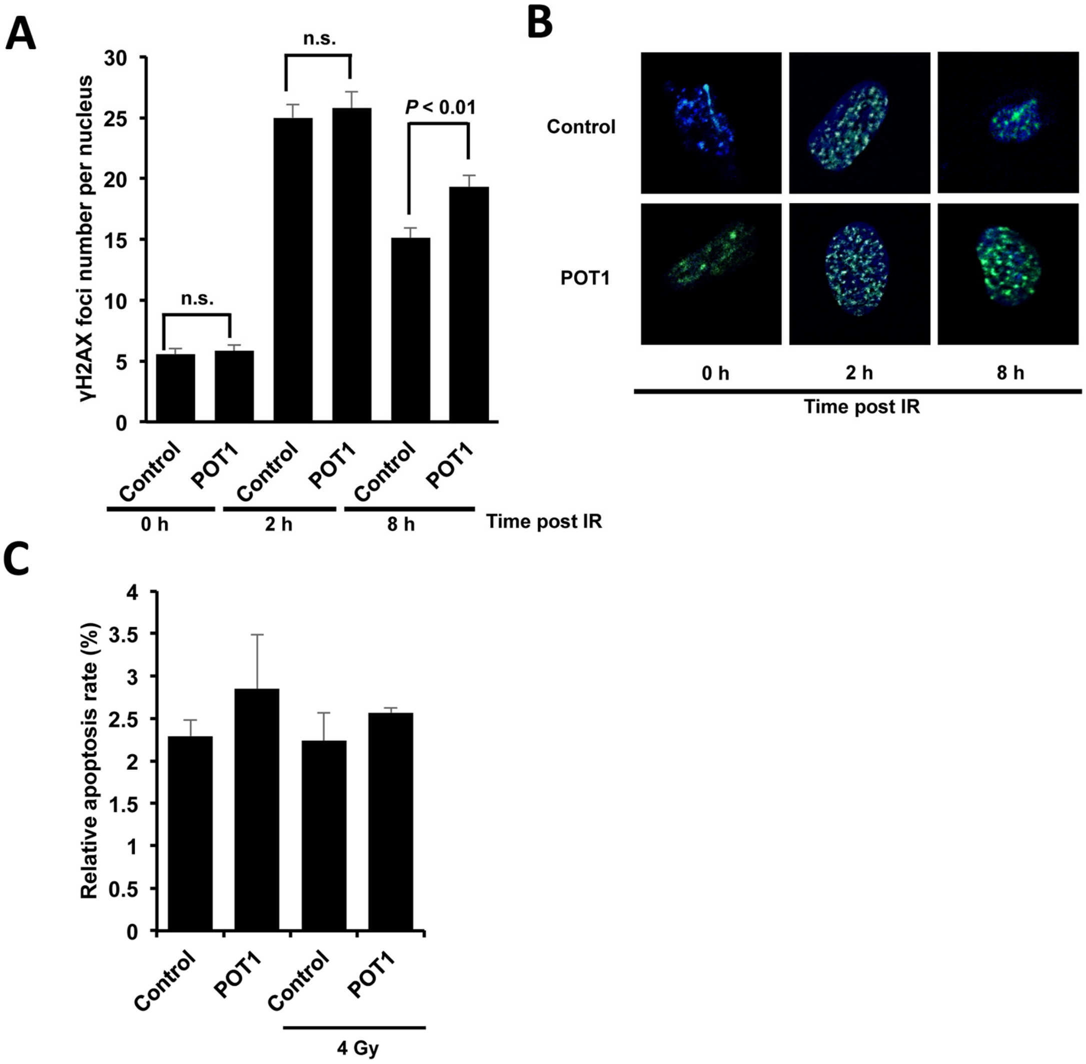

Supplementary Figure 4. POT1 overexpression suppresses the clearance of γH2AX foci in cells irradiated with X-ray, but it does not affect apoptotic rates. (**A**) At different time points post IR, cells were fixed, permeabilized and stained with an antibody against γH2AX. Then the number of foci per nuclear was counted on a fluorescent microscope (Nikon, Japan). (**B**) Representative pictures of γH2AX positive cells. (**C**) Cells with POT1 overexpressed were treated with IR at a dosage of 4 Gy. At 24 h post IR, cells were harvested for Annexin V staining. n.s., not significant.

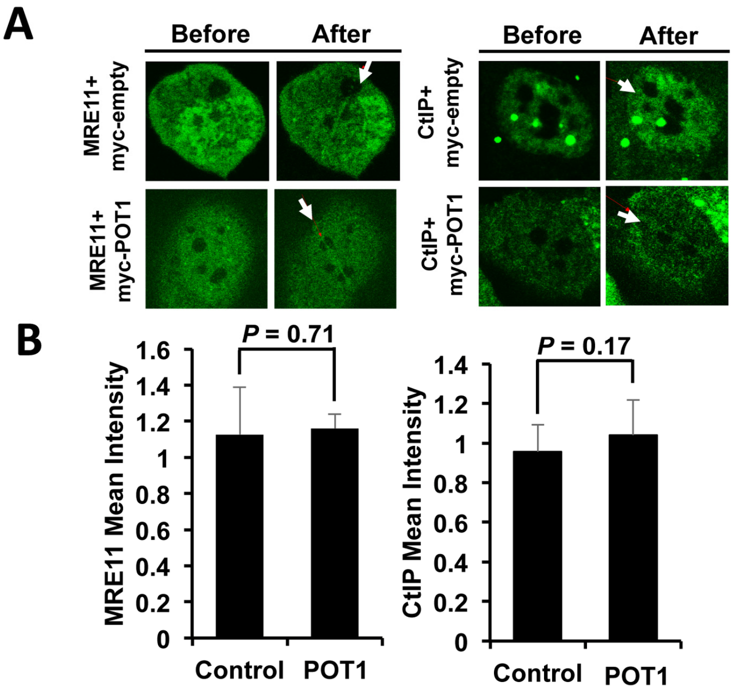

Supplementary Figure 5. POT1 overexpression has no significant effect on the recruitment of MRE11 and CtIP. (**A**) Representative pictures of the recruitment of MRE11 and CtIP to microirradiation induced DNA damage sites in the absence or presence of POT1 overexpression. (**B**) Statistic analysis indicates that no significant difference is observed between the two groups. The recruitment is quantified using the software of Leica LAS AF Lite.

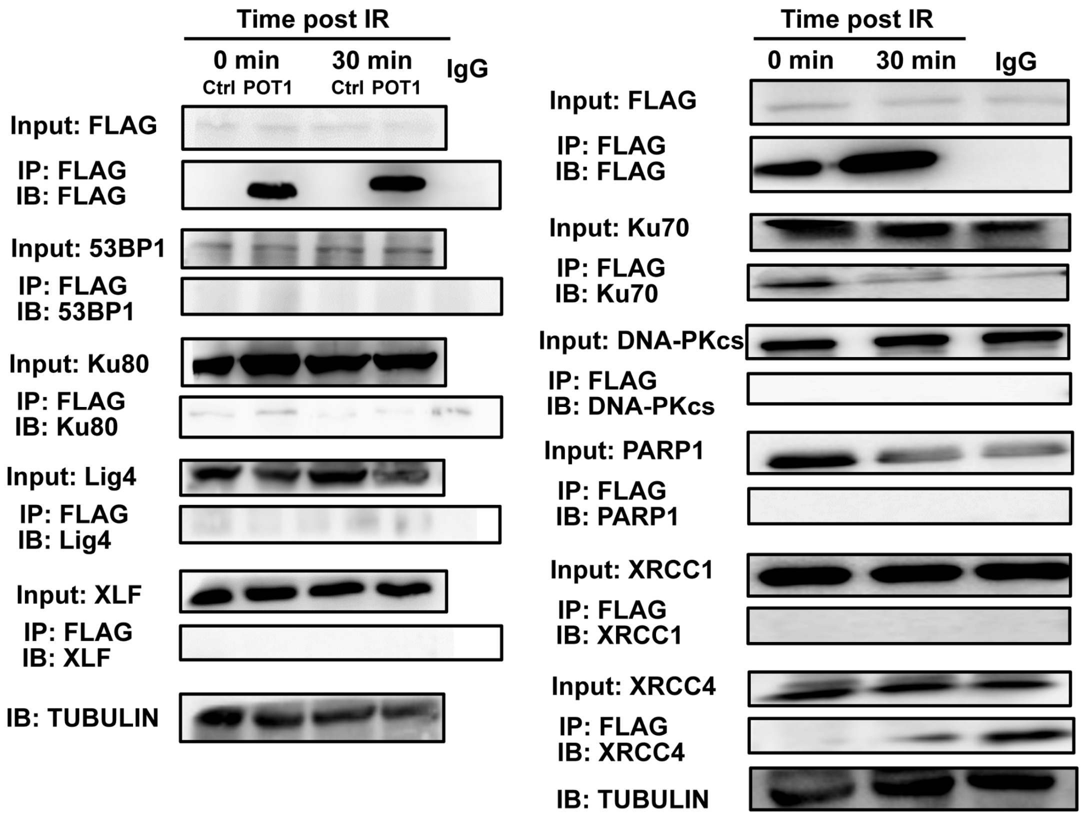

Supplementary Figure 6. Other NHEJ factors do not interact with POT1 in response to IR. The analysis is as described in Figure 3C.

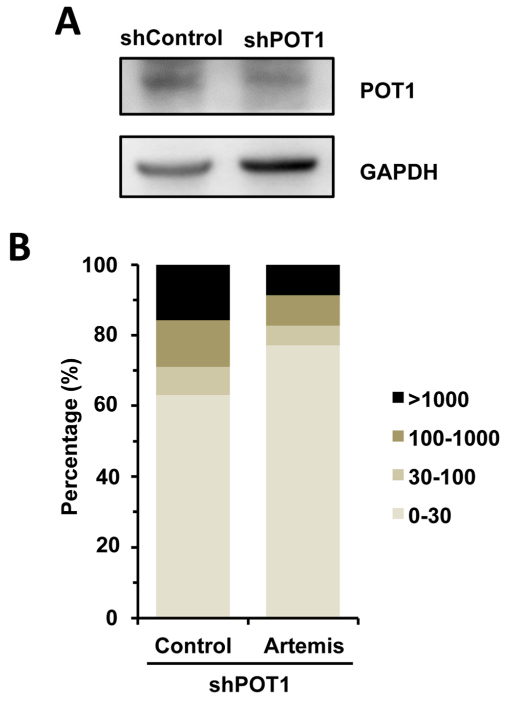

Supplementary Figure 7. Mildly knocking down POT1 partially impairs the stimulatory effect of Artemis overexpression on NHEJ fidelity. (**A**) Western blot analysis indicates that POT1 is mildly knocked down in HCA2-hTERT cells. (**B**) In cells with POT1 mildly knocked down, NHEJ fidelity was analyzed in the presence or absence of Artemis overexpression. The analysis method is the same as in Figure 2E. At least 40 clones were analyzed.
